# Impact of Time on Parameters for Assessing the Microstructure Equivalence of Topical Products: Diclofenac 1% Emulsion as a Case Study

**DOI:** 10.3390/pharmaceutics16060749

**Published:** 2024-06-01

**Authors:** Andreu Mañez-Asensi, Mª Jesús Hernández, Víctor Mangas-Sanjuán, Ana Salvador, Matilde Merino-Sanjuán, Virginia Merino

**Affiliations:** 1 Departamento de Farmacia y Tecnología Farmacéutica y Parasitología, Universidad de Valencia, Avda. Vicent Andrés Estellés s/n, Burjasot, 46100 Valencia, Spain; andreu.manez@uv.es (A.M.-A.); victor.mangas@uv.es (V.M.-S.); virginia.merino@uv.es (V.M.); 2 Instituto Interuniversitario de Investigación de Reconocimiento Molecular y Desarrollo Tecnológico (IDM), Universitat Politècnica de València, 46100 Valencia, Spain; 3 Departamento Física de la Tierra y Termodinámica, Universidad de Valencia, Avda. Vicent Andrés Estellés s/n, Burjasot, 46100 Valencia, Spain; m.jesus.hernandez@uv.es; 4 Instituto de Agroquímica y Tecnología de Alimentos (IATA-CSIC), Avda. Catedrático Agustín Escardino 7, Paterna, 46980 Valencia, Spain; asalvador@iata.csic.es

**Keywords:** topical bioequivalence, topical microstructure, topical rheology, internal structure of emulsions, regulatory aspects of formulations

## Abstract

The demonstration of bioequivalence proposed in the European Medicines Agency’s (EMA’s) draft guideline for topical products with the same qualitative and quantitative composition requires the confirmation of the internal structure equivalence. The impact of the shelf-life on the parameters proposed for internal structure comparison has not been studied. The objectives of this work were: (1) to quantify the effect of the time since manufacturing on the mean value and variability of the parameters proposed by the EMA to characterize the internal structure and performance of topical formulations of a complex topical formulation, and (2) to evaluate the impact of these changes on the assessment of the microstructure equivalence. A total of 5 batches of a topical emulgel containing 1% diclofenac diethylamine were evaluated 5, 14, and 23 months after manufacture. The zero-shear viscosity (η_0_), viscosity at 100 s^−1^ (η_100_), yield stress (σ_0_), elastic (G′) and viscous (G″) moduli, internal phase droplet size and in vitro release of the active ingredient were characterized. While no change in variability over time was detected, the mean value of all the parameters changed, especially the droplet size and in vitro release. Thus, combining data from batches of different manufacturing dates may compromise the determination of bioequivalence. The results confirm that to assess the microstructural similarity of complex formulations (such as emulgel), the 90% confidence interval limit for the mean difference in rheological and in vitro release parameters should be 20% and 25%, respectively.

## 1. Introduction

The development of generic medicines is key to society as it is one of the tools for ensuring access to medicines for most of the population and greatly contributes to the sustainability of healthcare systems. The characterization of the bioequivalence of immediate-release oral products with systemic action has a broad scientific, industrial, and regulatory consensus. Since the active substance in topical products exerts a local effect, leading to non-quantifiable levels in systemic circulation, and since sampling at the biophase is not feasible, the characterization of their bioequivalence represents a major challenge for stakeholders involved in both their development and evaluation. To address this issue, the FDA has issued product-specific, non-binding recommendations for the development of topical products. These recommendations outline specific approaches to be taken depending on the product, such as acyclovir and silver sulfadiazine creams, dapsone and bexarotene gels and benzyl alcohol lotion [1,2,3,4,5]. On the contrary, the European Medicines Agency (EMA) proposed more general rules through the publication of a draft guideline on the quality and bioequivalence of topical formulations in 2018, stating that two complex topical formulations could be considered bioequivalent when they have the same qualitative and quantitative composition, their microstructures are equivalent (extended to pharmaceutical equivalence) and their therapeutic equivalence is demonstrated by one of the accepted methods [6]. 

The microstructure of a topical formulation is determined by different properties, such as emulsions’ droplet size of the internal phase, the particle size of active ingredients in suspension, and rheological properties of the excipient. The EMA draft bioequivalence guideline recommends comparing the zero-shear viscosity (η_0_), viscosity at 100 s^−1^ (η_100_), yield stress (σ_0_), and elastic (G′) and viscous (G″) moduli to determine the internal structure equivalence of different formulations. Since these parameters are not routinely used in quality controls of topical formulations, knowledge is limited regarding which factors may affect their estimation when combining different batches of the same formulation. The time since manufacturing (within shelf-life) might be one of the factors that alter both the mean value and inter-batch variability, conditioning the determination of bioequivalence.

Since the EMA published the above-mentioned draft, various publications have analyzed properties related to the microstructure and their relationship with the release profile in various semi-solid formulations, some identical and others that are clearly different, to evaluate the validity of the proposed criteria. Several studies [7,8,9,10] showed that the large variability observed in the rheological parameters does not allow the conclusion of equivalence of the structure of products that are known to be the same. These studies were conducted with a small number of batches and replicates per batch, and the products tested did not necessarily have the same age since manufacture at the time of evaluation. It is therefore difficult to draw conclusions regarding the impact of the time since manufacture on the variability.

According to the EMA guidance, a minimum of three batches per formulation should be used for microstructure comparisons. The inter-batch variability of most of the parameters used to characterize the internal microstructure of formulations might, however, be sufficiently high to question the acceptable limits for equivalence proposed by the EMA [11]. Thus, as per the criteria proposed by the EMA, the internal microstructure of three batches of a product might not be considered equivalent to three batches of the same product. In a simulation-based study comparing 3 batches with 12 replicates per batch and no differences in the mean value of the parameter, the 15% intra- and 2.5% inter-batch coefficient of variation (CV) microstructure equivalence would only be concluded in 80% of the comparisons, while a 2.5% intra- and 15% inter-batch CV equivalence would only be considered in 55% of the comparisons. A total of 6 batches with 12 replicates per batch would be required to establish equivalence within the proposed limits of the EMA guidance when 2.5–5% differences in the mean value test/reference ratios exist. When the intra- or inter-batch variability exceeds 10%, a higher number of batches or replicates per batch would be required [7,11,12,13]. All these situations are not unlikely due to variations in the manufacturing process or raw material origin. In recent years, the implementation of the components of Quality by Design (QbD) in the manufacture of topical products has been promoted [14,15,16]. Hopefully, the consideration of rheological parameters at this stage will imply lower variations between batches [17,18]. Nevertheless, at this moment, the regulatory framework is very demanding, but the rotation of topical products in the market is low; thus, having sufficient batches of the same formulation manufactured at the same time poses a challenge for bioequivalence assessment.

The objectives of this work are (i) to quantify the impact of the time elapsed since manufacturing within the shelf-life of a batch on the mean value and variability of rheological parameters selected in the EMA document to represent the internal structure of a complex topical formulation (η_0_, η_100_, σ_0_, G′ and G″ and internal phase droplet size of an emulsion) and its performance (in vitro release) and (ii) to evaluate the impact of such changes on the criteria proposed by the EMA for establishing the microstructure equivalence of topical formulations.

## 2. Materials and Methods

A total of 5 batches of a commercialized topical emulgel formulation containing 1% (11.6 mg/g) diclofenac diethylamine (Kern Pharma, Terrassa, Barcelona, Spain) were evaluated. According to the technical data sheet [19], the formulation includes as excipients: propyleneglycol (E-1520), isopranolol, diethylamine, liquid paraffin, polyoxyethylene alkyl ether, carbomer 934P NF, coco-caprylate/caprate, perfume cream 45399 and purified water. All had the same expiry date. The samples were stored at between 20 and 25 °C.

The samples were analyzed 5, 14, and 23 months after their manufacturing (as soon as samples were available, before they expired and at an intermediate time point). At each analysis time, 12 replicates of each batch were evaluated to determine the parameters described below. 

### 2.1. Rheological Analysis

The measurements were performed with a controlled stress rheometer (RheoStress RS1, Thermo Scientific-Haake^®^, Karlsruhe, Germany), using the RheoWin 4.0 software. Prior to the measurements, the samples were kept at rest at least 600 s after loading to ensure stress relaxation and temperature equilibrium. Measurements (*n* = 12 replicates per batch) were performed at 20 °C (storage conditions) using serrated parallel plates (35 mm diameter, 1 mm gap). The following rheological measurements were performed.

#### 2.1.1. Hysteresis Loops 

The hysteresis loops were measured in control rate mode. Up and down flow curves from 1 s^−1^ to 100 s^−1^ were performed (10 s/step, 20 steps in log distribution). Down curves were recorded after 60 s at the maximum shear rate. The relative thixotropic area, S_R_, was calculated to characterize the thixotropy. The S_R_ (%) was estimated from the area enclosed by the upward (S_U_) and the downward (S_D_) curves as [(S_U_-S_D_)/S_U_]·100 [20]. Numerical integration was performed with Kaleida graph 4.03 software (Synergy Software).

#### 2.1.2. Flow Curves

Stepped flow curves were performed in controlled stress mode (60 s per step in logarithmic distribution). The shear stress range was chosen to obtain shear rates ranging from very low values (approx. 10^−5^ s^−1^) to approx. 100 s^−1^. The simplified Carreau model (Equation (1)) was satisfactorily fitted to the viscosity values as a function of the shear rate data [21] with Kaleida graph 4.03 software (Synergy Software):

(1) $$\eta =\frac{\eta_{0}}{{\left(1+{\left(\frac{\dot{\gamma }}{{\dot{\gamma }}_{c}}\right)}^{2}\right)}^{s}}$$

where η_0_ is the zero-shear viscosity, ${\dot{\gamma }}_{c}$ the critical shear rate, and s the shear thinning index related to the slope of the viscosity curve for high shear rates in a double-log scale. From the curve fits obtained, the zero-shear viscosity (η_0_) and the viscosity corresponding to 100 s^−1^ were calculated (η_100_) and used to compare batches. 

#### 2.1.3. Small-Amplitude Oscillation Sweep Tests

Oscillatory sweep tests were performed at 1 Hz, varying the stress amplitude from 0.5 to 20.0 Pa (10 point/decade in log distribution). The rheological viscoelastic parameters determined were the elastic (G′) and viscous (G″) moduli in the linear viscoelastic region (LVR) and the yield stress (σ_0_). The values of both viscoelastic moduli (G′ and G″) correspond to the average of the constant values in the plateau region of the LVR. The yield stress was estimated as the stress value at which the G′ curve begins to deviate from the constant values in the LVR (10% tolerance).

### 2.2. Droplet Size Measurement

The size of the internal phase droplets was measured using an optical microscope Leica DMR (40×) (L’Hospitalet de Llobregat, Spain), image capture system DFC450C and LAS Software V. 4. Approximately 120 droplets were manually measured for each formulation batch at each of the sampling times.

### 2.3. In Vitro Release Test (IVRT)

In vitro release tests through Tuffryn membranes (hydrophilic polyethersulfone 0.45 μm pore, 25 mm diameter, 140 μm thickness (PALL Corporation, Ann Arbor, MI, USA) were conducted according to the USP general chapter <1724> [22]. The experiments were performed by employing vertical Franz-type diffusion cells with an effective area available for diffusion of 0.79 cm^2^ and a receiver compartment capacity of approximately 6 mL (Vidrafoc, Barcelona, Spain). The membrane’s inertness toward diclofenac diethylamine and validity for the purpose of the assay were previously confirmed [12]. The membranes were pre-soaked with receptor solution (50 mM potassium phosphate buffer, pH 7.4) for 30 min. To properly define the release rate profile, an infinite dose (300 nominal mg) of diclofenac emulgel was uniformly applied in the dosage chamber. The receptor solution within the chamber was stirred at 200 rpm and the temperature was maintained at 32 ± 1 °C. Sink conditions were ensured during the assay.

At least 6 samples were collected within 6 h and stored at −20 °C until analysis. Samples were taken at 0.5, 1, 2, 3, 4 and 6 h. The amount of drug released per unit membrane area was plotted versus the square root of the time. The release rate of diclofenac diethylamine was calculated from the slope of this plot using Microsoft Excel 365. The total amount released at the end of the test (A_6h_) was also determined. 

### 2.4. Drug Quantification 

The diclofenac diethylamine samples were analyzed using high-performance liquid chromatography (HPLC) with UV detection. The analytical method was previously validated. Samples were injected into a Brisa LC^2^ C18 (Teknokroma) column (5 µm particle size, 4.6 × 150 mm). The mobile phase consisted of 70% acetonitrile and 30% acetate buffer (700 mM, pH 3.0). The flow rate was 1 mL/min and the samples were analyzed at 280 nm; under these conditions, the retention time of diclofenac diethylamine was 3 min. The samples’ concentration was calculated by interpolation using a calibration curve. 

The chromatographic method was validated following the recommendations of the International Council on Harmonisation in terms of the linearity, precision (intra- and interday), accuracy, limit of detection and quantification, specificity, interval and robustness [23]. The lower limit of quantification of diclofenac was 2.03 μg/L.

### 2.5. Statistical Analysis and Data Comparison

An exploratory numerical evaluation was conducted by calculating the mean value and the coefficient of variation (CV%) of each parameter. 

The Shapiro–Wilk test was performed to evaluate whether the parameters followed a normal distribution. Parameters not following a normal distribution were log-transformed for further comparison. The differences between batches and sampling times were evaluated using an ANOVA test, followed by the post hoc Tukey test. The significance level for these tests was set at α = 0.05.

The evaluation of equivalence was established using the criteria proposed by the EMA (Committee for Medicinal Products for Human Use (CHMP) 2018). The 90% confidence interval (CI) of the geometric mean ratio (GMR) between the test (longer time since manufacturing) and reference formulation (shorter time since manufacturing) of each parameter was calculated. Parameters were considered equivalent (compliant) if the 90% CI of the GMR of the rheological and IVRT parameters lay within the limits of 90–111.11%. 

The overall variability among the different parameters was determined by a principal component analysis (PCA). 

The analyses were performed using R (V. 4.0.4) and R-studio (V. 1.4.1106) software and Microsoft Excel 365 and the PCA using XlStat (2019, Addinosoft, Barcelona, Spain).

## 3. Results and Discussion

### 3.1. Parameter Evolution during Shelf Life

The rheological properties, the droplet size, and the active ingredient release of a complex semisolid formulation (emulgel) have been evaluated throughout the shelf-life of the pharmaceutical product to assess its impact on the mean value, the dispersion and the declaration of equivalence. The batches used for this analysis were manufactured by the same company with the same technical requirements and were approved for their commercialization (i.e., they were considered equivalent). 

The evaluation of the shear stress versus shear rate in both the upward and downward directions was coincident, resulting in an area enclosed by them (S_R_) of zero. Thus, the formulation did not exhibit any hysteresis loops and no thixotropic behavior was detected.

**Figure 1:**
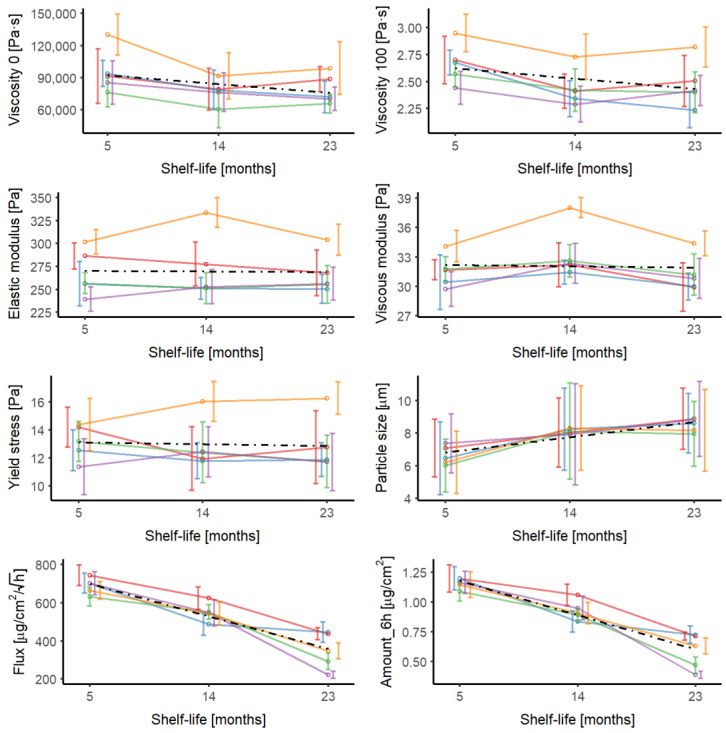
Longitudinal evolution of the parameters across the shelf-life (5, 14, and 23 months) of the formulation. The mean values with the standard deviation of each batch (*n* = 12) are represented as empty-colored circles and colored solid lines (red: R030, blue: R031, green: R032, purple: R033, orange: R034). The dashed black line corresponds to the mean value of the parameter at each time tested (*n* = 60).

**Figure 2:**
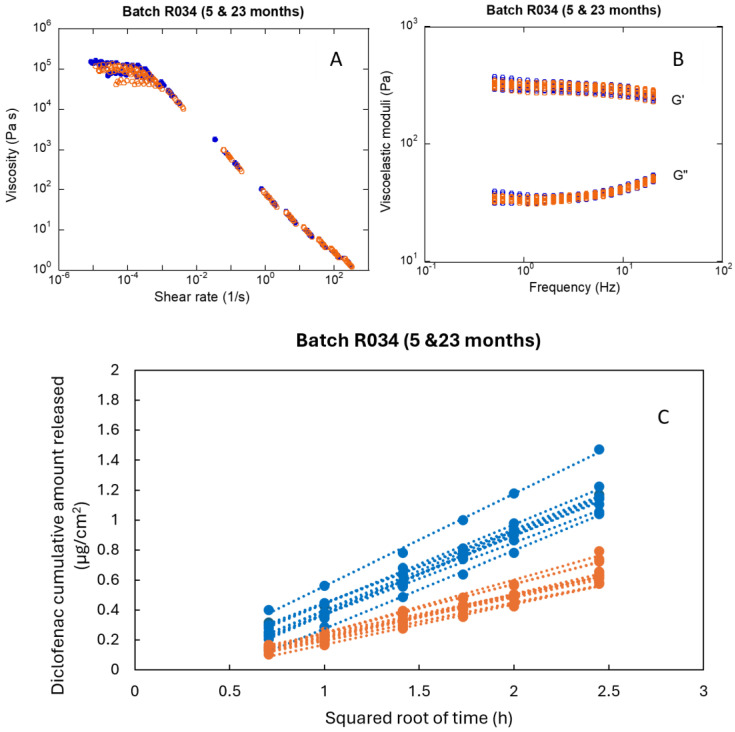
Flow curves (**A**), oscillatory tests (**B**), and in vitro release profiles (**C**) of Batch R034 at 5 (blue) and 23 months (orange). *n* = 12 per time plot.

Figure 1 shows the distribution of the parameters at 5, 14, and 23 months after the manufacturing date for each batch. The results are presented as the mean value and standard deviation of the parameters per batch. As a reference, the global mean value of the parameter at each time point has been represented (black dashed line). The longitudinal evolution of the intra-batch, inter-batch, and total variability of each parameter is shown in Table 1. The mean values of the parameters for each batch and the global mean are summarized in Table S1 in supplementary material. The flow curves, the oscillatory tests, and the individual values of IVRT flux, corresponding to the first and last analyses (5 and 23 months) of Batch R034 (Figure 2), illustrate the role of intra-batch variability.

The zero-shear viscosity shows a very high variability, both within and between batches, higher than 15% in most of the analyses (Table 1, Figure 1). Since the zero-shear viscosity corresponds to the response of the sample to extremely low stresses (reflecting the consistency of the sample at rest and, thus, of its internal structure), it is calculated as the resistance of the sample when applying shear rates around 0.0001 s^−1^. This parameter is very sensitive to any deviation in the measuring protocol, environmental conditions, and inhomogeneities in the sample. Despite extreme care being taken to control the test conditions, it is impossible to eliminate the dispersion of the replicates. These differences are minimized in the rheological log-log scale but are detected with statistical tests (Figure 2B). Although the intra-batch coefficient of variation of the yield stress is slightly lower in most measurements (around 10%), it increases >18% in some. The inter-batch variability does not correlate with the time, except for σ_0_, which increases over time (Table 1). The intra-batch variability of both IVRT parameters, flux and total amount of diclofenac released at 6 h, is moderate (<8.5%). The variability of both parameters increased over time. The droplet size is also associated with high total variability due to the high intra-batch variability (25–30%), although the inter-batch variability is lower than 8%, which could be because the measurements were performed manually. In contrast, the viscosity at 100 s^−1^, as well as the elastic and viscous moduli, exhibit moderate variabilities. The intra-batch, inter-batch and total variability of these parameters is <10%, except for G′ at 14 months, which is slightly higher. 

The mean value of some parameters varied over time (Figure 1). An ANOVA test was used to determine the statistical significance of the differences in the parameters in batches with different times since manufacturing. No significant differences were found in the yield stress and elastic modulus parameters, but statistically significant differences were observed for the other parameters (Table 2). The impact of the shelf-life on the mean value of the parameters was also observed within each batch (Table S2). The viscous modulus exhibited an initial increase followed by a subsequent decrease over time. The elastic modulus and yield stress were obtained from the same oscillatory tests but did not exhibit the same trend of change over time, either for the mean value of the parameter or the variability. These differences have statistical but not physical significance due to the inherent variability of rheological measurements. The zero-shear viscosity and viscosity at 100 s^−1^ dropped between 5 and 14 months, not changing thereafter. Within the 9 months from the first reading, the average droplet size increased by 21% (from 7 to 8.45 μm), remaining constant afterwards (no statistically significant difference as per the ANOVA test). This suggests that the coalescence phenomenon occurred in the first months of the formulation. The droplet size increases and the viscosity decreases (the first with a higher significance) over time. The relationship between both parameters is in alignment with what has been observed for emulsions [17,24]. After 14 months, the zero-shear viscosity and viscosity at 100 s^−1^ decreased to 91% and 85%, respectively, of their original values. 

The evolution of the rheological parameters over time has been observed in other topical products. Capsaicin emulsions decreased in viscosity at 300 s^−1^, yield stress, and complex modulus over 10 months, while the ratio between G′ and G″ remained constant [10]. In contrast, we only observed a decrease in viscosity. The intra-batch and inter-time variabilities of the capsaicin emulsions were not excessively high, but given the changes in the mean values of the parameters experienced over time, the variability increases when batches of different shelf-lives are combined [10]. In bifonazole creams, no clear relationship between the changes in yield stress and the shelf-life were found and differences in their rheological parameters did not correlate with changes in the release of the active ingredient [8]. In contrast to these findings, our IVRT results showed a marked decrease in the fluxes obtained over time, mainly between the second and the third measurements. Batch R031 was a notable exception, with the strongest decline occurring in the first few months (Figure 1). 

**Figure 3:**
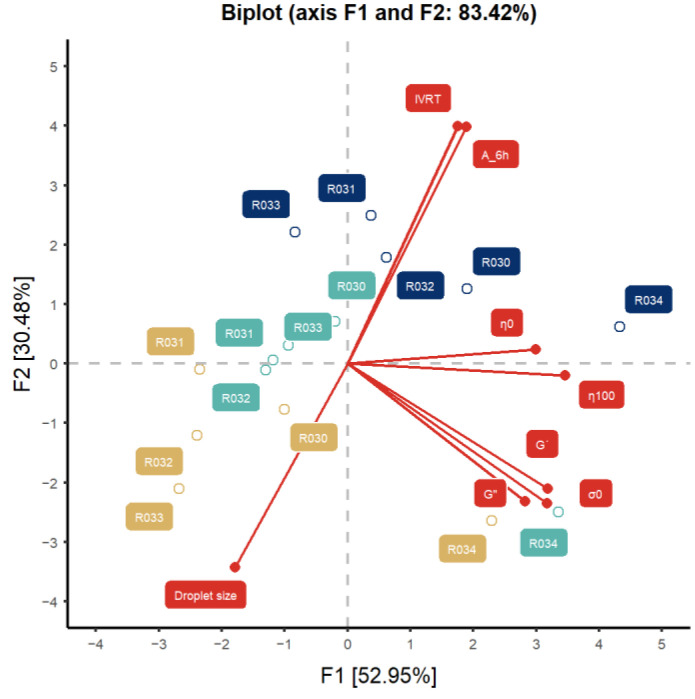
Principal component analysis (PCA) of the parameters studied. G′: elastic modulus, G″: viscous modulus, σ_0_: yield stress, IVRT: in vitro release test flux, A_6h: total amount released at the end of 6 h IVRT. Brown, green, and blue labels and dots represent data from 5, 14, and 23 months, respectively.

A two-dimensional plot accounted for 83.43% of the total variance (Figure 3). The first dimension (52.95% of the variability) positively related to the rheological parameters (η_0_, η_100_, G′, G″ and σ_0_) and the in vitro release parameters (flux [IVRT] and total amount released after 6 h assay [A_6h_]) and negatively related to the droplet size. The second dimension (30.48% of variability) positively related to the IVRT flux and A_6h_ and negatively related to the droplet size, also showing an inverse correlation between the emulsion’s droplet size and the release rate from the formulation (180° angle). The first dimension differentiated between samples measured at 5 months and those measured at 14 and 23 months. The samples at 5 months (right quadrant) presented higher rheological parameters and in vitro release parameters, whereas samples measured at 23 months had a higher droplet size. Similar observations were made for all the batches except for Batch 34, for which measurements at the three times since manufacturing are related to the rheological and in vitro release parameters.

### 3.2. Impact of Parameters Evolution on Equivalence Contrast

**Figure 4:**
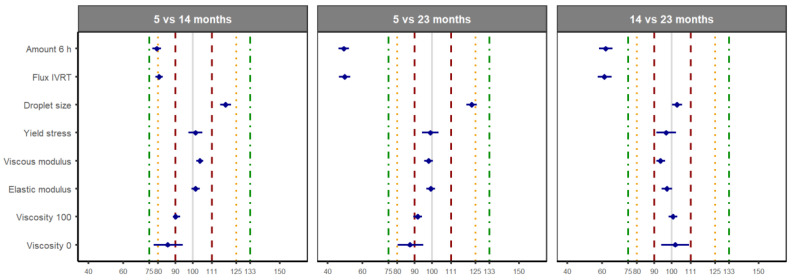
The 90% CI of the difference in the means (log-transformed data) for the indicated parameters. Limits for the 90% CI have been included as dashed red (10%), dashed orange (20%), and dashed green (25%) as references. The residual variance was obtained from a one-way ANOVA test. *n* = 60 per time point.

The criteria proposed by the EMA were applied to analyze the differences in the parameters determining the structure of the topical formulation over time. Comparisons were performed globally, combining the data of the five batches, to determine if the differences in the mean value of each parameter observed over time would have an impact on bioequivalence decision-making. The residual variance for the calculation of the 90% CI was obtained from a one-way ANOVA test considering the time factor. Based on our findings and as per the EMA criteria, as the time since manufacturing passes, a formulation may no longer be equivalent to itself (Figure 4). Remarkably, the equivalence of the rheological parameters and droplet size is observed between 14 and 23 months, but not between 14 and 5 months or between 23 and 5 months (see Table 2 and Figure 4 [red lines]). The droplet size, zero-shear viscosity and viscosity at 100 s^−1^ were equivalent when comparing 23 vs. 5 months if the limits were extended to 20% (Figure 4, orange line). When the 90% CI limit was extended to 25%, equivalence was achieved when comparing all the parameters, including those of the IVRT obtained at 14 months with those at 5 months (Figure 4, green lines). The zero-shear viscosity also required the extension of the limits to 25% to accomplish equivalence in the comparison of 14 vs. 5 months. Extension of the limits to 25% did, however, not conclude the equivalence of the IVRT parameters when comparing 23 vs. 5 or 23 vs. 14 months (Figure 4). These results suggest that structural changes in emulgels (and probably also in emulsions) occur shortly after manufacturing, with the equivalence being compromised when batches with different shelf-lives are combined. The data were reanalyzed using a two-way ANOVA to calculate the residual variance required for the 90% CI, including the batch and time as factors. The 90% CI obtained by this method were narrower, with the 90% CI of the zero-shear viscosity being within the 20% limit in the three comparisons performed (Figure S1). 

Finally, to confirm that the differences detected in the analyses can be attributed to the effect of time, the values of the rheological and IVRT parameters obtained in batches with the same time since manufacturing were compared. As recommended by the EMA, the comparisons were performed using three vs. three batches. Since only five of the required six batches of formulation were available for testing, the data of one batch were duplicated and used in both the reference and test groups. All 18 possible combinations of 3 vs. 3 were tested (Table 3). While confirmation of equivalence would have been expected for all the combinations, when considering the 10% limit for the 90% CI of the differences in GMR ratios, the G″ was the only parameter equivalent for 100% of combinations and the η_0_ was not equivalent for any combination. The other parameters showed differences depending on the time elapsed since manufacturing (5, 14, or 23 months), and the number of parameters reaching equivalence decreased with time. When the limit of the 90% CI was extended to 20%, all the parameters except the η_0_ were equivalent at 5 and 14 months. Small differences in the manufacturing process could translate into differences between the batches. The parameters determined by the IVRT test failed to demonstrate equivalence at 23 months, even when a 20% range was considered. When the range was extended to 25%, as suggested in the SUPAC guideline, the IVRT parameters were equivalent in all the comparisons made [22]. Altogether, these results confirm the importance of the time elapsed since manufacturing for the assessment of structural equivalence, reinforcing the need to widen the limits of the 90% CI proposed in the EMA draft guideline to compare the sameness of the microstructure of topical products.

## 4. Conclusions

This study shows that the passage of time since manufacturing (within its shelf-life) changes five out of seven parameters used to characterize the equivalence of a product’s structure. The droplet size and IVRT parameters are more sensitive to the impact of time than the rheological parameters. The changes in the variability of the parameters are quite uniform, occurring to a similar extent in all the batches, suggesting that the quality of all the batches is homogeneous and the time has a very similar effect on all the batches. 

The evolution in the value of the parameters is important one two levels, compromising the decision of equivalence. (1) When comparing batches of different ages, the changes in the mean value of the parameters may result in differences in the ratio test/reference. This can shift the 90% CI outside the limit. (2) When combining batches with different manufacturing dates, changes in the mean value can increase the inter-batch and total variability of the parameters being compared. Our results show that a 90% CI limit for the mean difference of 20% for the rheological parameters and 25% for the IVRT parameters might be more appropriate to consider the equivalence of the microstructure of complex formulations such as the evaluated emulgel. Moreover, the η_0_ should be excluded from the equivalence comparison as its determination is subject to large variability independently of the age of the formulation. 

## Figures and Tables

**Table 1:** Intra-batch (*n* = 12), inter-batch (*n* = 5), and total variability (*n* = 60) of indicated parameter, expressed as the variation coefficient in percentage (CV%), in samples analyzed 5, 14, and 23 months after manufacturing. η_0_: zero-shear viscosity, η_100_: viscosity at 100 s^−1^, G′: elastic modulus, G″: viscous modulus, σ_0_: yield stress, IVRT: in vitro release test, A_6h_: total amount released at the end of 6 h IVRT.

Parameter	Time (Months)	CV % of the Parameter						
		R030	R031	R032	R033	R034	Inter-Batch	Total
η_0_	5	30.4	13.5	17.9	25.8	14.7	23.5	29.0
	14	23.8	22.5	28.1	25.1	23.8	13.9	26.8
	23	12.9	20.4	13.8	15.8	25.8	17.6	24.6
η_100_	5	7.8	4.3	4.6	6.1	5.8	7.0	8.6
	14	6.5	7.1	8.2	7.1	7.6	7.0	9.5
	23	9.4	7.2	8.0	5.7	6.5	8.9	10.8
G′	5	4.9	9.9	4.4	5.5	4.4	10.3	11.0
	14	8.7	4.6	6.8	7.4	4.8	12.5	13.0
	23	9.4	6.4	8.2	7.0	5.5	9.0	10.8
G″	5	3.2	9.4	3.9	5.7	4.7	6.1	7.7
	14	7.1	3.8	5.1	6.4	2.6	8.4	9.0
	23	8.4	4.6	6.8	6.6	3.7	6.6	8.4
σ_0_	5	10.5	11.9	10.7	18.2	13.0	10.2	15.4
	14	17.6	10.6	17.5	14.0	8.9	11.9	17.1
	23	20.5	10.2	15.8	18.2	7.2	15.1	19.6
droplet size	5	23.9	32.3	25.6	23.6	28.1	7.8	27.6
	14	25.3	29.6	33.9	38.2	29.8	2.8	31.6
	23	21.3	21.2	24.6	25.2	30.1	5.0	24.9
IVRT Flux	5	7.4	7.4	7.8	8.4	4.3	5.9	8.9
	14	9.3	11.3	7.0	12.2	11.1	8.6	12.6
	23	7.45	11.7	14.7	8.2	11.45	26.9	26.5
A_6h_	5	9.34	8.21	7.51	7.80	4.56	4.0	8.3
	14	8.25	10.59	5.98	10.43	10.02	9.9	12.6
	23	4.79	9.73	14.39	7.84	10.58	14.4	24.8

**Table 2:** Results of the ANOVA post hoc Tukey tests for the comparison of parameters over time and 90% confidence interval of the difference of the means of the parameters for the comparisons done (5 vs. 14, 14 vs. 23, and 5 vs. 23 months) the * Statistically significant differences (*p* < 0.05), *n* = 60 vs. 60. NC = not compliant bioequivalence as per the EMA criteria; C = compliant bioequivalence as per the EMA criteria. NS: not significant.

Parameter	Compared Groups	ANOVA	EMA CI 90%	Status
η_0_	5 vs. 14	*	85.5 (77.5–94.3)	NC
	14 vs. 23	NS	102.0 (94.0–110.0)	C
	5 vs. 23	*	87.2 (80.2–94.9)	NC
η_100_	5 vs. 14	*	90.1 (89.2–92.9)	NC
	14 vs. 23	NS	100.7 (98.3–103.3)	C
	5 vs. 23	*	91.7 (89.2–94.3)	NC
G’	5 vs. 14	NS	101.6 (99.3–104.0)	C
	14 vs. 23	NS	97.2 (94.3–100.2)	C
	5 vs. 23	NS	99.2 (96.7–101.7)	C
G”	5 vs. 14	*	104.2 (102.2–106.0)	C
	14 vs. 23	*	93.6 (91.2–96.2)	C
	5 vs. 23	NS	97.9 (95.5–100.4)	C
σ_0_	5 vs. 14	NS	101.5 (97.7–105.5)	C
	14 vs. 23	NS	96.8 (91.4–102.5)	C
	5 vs. 23	NS	98.9 (94.1–103.8)	C
Droplet size	5 vs. 14	*	118.94 (115.8–122.2)	NC
	14 vs. 23	NS	103.1 (100.4–106.0)	C
	5 vs. 23	*	122.7 (119.7–125.7)	NC
IVRT Flux	5 vs. 14	*	80.7 (78.7–82.9)	NC
	14 vs. 23	*	61.3 (57.5–65.5)	NC
	5 vs. 23	*	49.6 (46.5–52.8)	NC
A_6h_	5 vs. 14	*	79.29 (76.77–81.89)	NC
	14 vs. 23	*	61.95 (58.17–65.97)	NC
	5 vs. 23	*	49.12 (46.26–52.15)	NC

**Table 3:** Comparisons (3 vs. 3 batches, *n* = 36 vs. *n* = 36) of the parameters at the same time since manufacturing by the means of the 90% CI of the difference in the means of the parameters (log-transformed data).

	INTERVAL		η_0_	η_100_	G′	G″	σ_0_	Droplet Size	IVRT Flux	A_6h_
5 months	90–111	*n*	0/18	18/18	11/18	18/18	5/18	7/18	18/18	18/18
		%	0	100	61.11	100	27.77	38.88	100	100
	80–125	*n*	5/18	18/18	18/18	18/18	18/18	18/18	18/18	18/18
		%	27.77	100	100	100	100	100	100	100
	75–133	*n*	13/18	18/18	18/18	18/18	18/18	18/18	18/18	18/18
		%	72.22	100	100	100	100	100	100	100
14 months	90–111	*n*	0/18	18/18	3/18	18/18	4/18	0/18	10/18	11/18
		%	0	100	16.66	100	22.22	0	55.55	61.11
	80–125	*n*	11/18	18/18	18/18	18/18	18/18	18/18	18/18	18/18
		%	61.11	100	100	100	100	100	100	100
	75–133	*n*	18/18	18/18	18/18	18/18	18/18	18/18	18/18	18/18
		%	100	100	100	100	100	100	100	100
23 months	90–111	*n*	0/18	12/18	11/18	18/18	1/18	4/18	0/18	5/18
		%	0	66.66	61.11	100	5.55	22.22	0	27.77
	80–125	*n*	11/18	18/18	18/18	18/18	18/18	18/18	7/18	17/18
		%	61.11	100	100	100	100	100	38.88	94.44
	75–133	*n*	16/18	18/18	18/18	18/18	18/18	18/18	18/18	18/18
		%	88.88	100	100	100	100	100	100	100

## Data Availability

The original contributions presented in the study are included in the article/Supplementary Material, further inquiries can be directed to the corresponding author.

## Supplementary Materials

The following supporting information can be downloaded at https://www.mdpi.com/article/10.3390/pharmaceutics16060749/s1, Figure S1: The 90% CI of the difference in the means of the parameters (two-way ANOVA test); Table S1: Mean value of the parameters measured; Table S2: Comparison of the analyzed rheological and performance parameters at the three time points since manufacturing, as evaluated batch by batch.
