# Supplementary material for: Impact of Time on Parameters for Assessing the Microstructure Equivalence of Topical Products: Diclofenac 1% Emulsion as a Case Study
Source: Pharmaceutics. 2024 Jun 1;16(6):749. doi: 10.3390/pharmaceutics16060749 (PMC11207613; doi:10.3390/pharmaceutics16060749)
Supplement: Supplementary file 1 [file pharmaceutics-16-00749-s001.zip › pharmaceutics-2971139-supplementary.pdf]

IMPACT OF TIME ON PARAMETERS FOR ASSESSING MICROSTRUCTURE EQUIVALENCE OF TOPICAL PRODUCTS

Andreu Mañez Asensi, Mª Jesús Hernández, Víctor Mangas-Sanjuán, Ana Salvador, Matilde Merino-Sanjuán and Virginia Merino

SUPPLEMENTARY MATERIAL

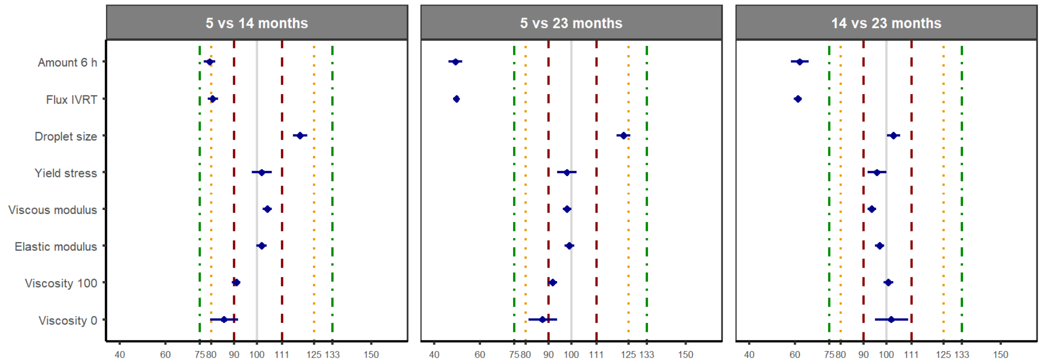

Figure S1. 90% CI of the difference in means (log-transformed data) of the indicated parameters. Limits for the 90% CI have been included as dashed red (10%), dashed orange (20%), and dashed green lines (25%). The residual variance was determined by a two-way ANOVA test.

Table S1. Mean value of the parameters measured: zero-shear viscosity (η_0), viscosity at 100 s^{-1} (η_{100}), elastic (G') and viscous modulus (G''), yield stress (σ_0), droplet size, flux and total amount of diclofenac accumulated in the receptor chamber at 6 h (A_{6h}) of *in vitro* release test (IVRT) at the 3 timepoints since manufacturing analyzed.

Parameter	Time (months)	Mean values of the parameter for the batch					
		R030	R031	R032	R033	R034	Global Mean
η_0 (Pa·s)	5	84257	89768	75320	78885	128558	91358
	14	82089	82605	61962	71966	89738	77672
	23	91651	72272	64744	69248	95420	78667
η_{100} (Pa·s)	5	2.80	2.65	2.58	2.49	2.98	2.7
	14	2.40	2.36	2.39	2.35	2.76	2.46
	23	2.50	2.26	2.37	2.45	2.84	2.48
G' (Pa)	5	288.11	245.8	255.78	239.46	302.69	266.37
	14	270.75	251.38	251.89	251.66	330.68	272.31
	23	264.05	245.73	250.05	253.34	304.51	263.54
G" (Pa)	5	31.66	29.41	31.80	30.15	34.42	31.49
	14	31.00	31.22	32.27	31.73	37.89	32.97
	23	29.26	29.51	30.55	30.60	34.35	30.85
σ_0 (Pa)	5	13.65	12.26	13.11	10.99	14.41	12.89
	14	12.49	12.0	12.36	12.75	15.93	13.19
	23	12.6	11.7	11.89	11.26	16.03	12.7
Droplet size (μm)	5	7.37	6.90	6.31	7.85	6.61	7.0
	14	8.34	8.33	8.70	8.18	8.64	8.45
	23	8.88	8.53	8.07	9.14	8.28	8.58
IVRT Flux (μg/cm²/√h)	5	729.56	702.65	639.37	717.97	652.07	688.9
	14	638.73	507.05	546.48	558.89	548.41	559.91
	23	430.50	456.40	289.70	228.90	362.78	353.67
A_{6h}(μg/cm²)	5	1.21	1.19	1.10	1.19	1.13	1.16
	14	1.08	0.84	0.89	0.94	0.90	0.93
	23	0.70	0.74	0.47	0.39	0.64	0.59

Table S2. Comparison of rheological and performance parameters at the 3 time points since manufacturing analyzed, evaluated batch by batch. Zero-shear viscosity (η_0), viscosity at 100 s^{-1} (η_{100}), elastic (G') and viscous modulus (G''), yield stress (σ_0), droplet size, flux and total amount of diclofenac accumulated in the receptor chamber at 6 h (A_{6h}) of *in vitro* release test (IVRT). *Denotes statistical differences ($p<0.05$).

Parameter	Groups compared	R030	R031	R032	R033	R034
η_0	5 vs 14	p>0.05	p>0.05	*	p>0.05	*
	5 vs 23	p>0.05	*	*	p>0.05	*
	14 vs 23	p>0.05	p>0.05	p>0.05	p>0.05	p>0.05
η_{100}	5 vs 14	*	*	p>0.05	p>0.05	*
	5 vs 23	*	*	p>0.05	p>0.05	p>0.05
	14 vs 23	p>0.05	p>0.05	p>0.05	p>0.05	p>0.05
G´	5 vs 14	p>0.05	p>0.05	p>0.05	p>0.05	*
	5 vs 23	*	p>0.05	p>0.05	p>0.05	p>0.05
	14 vs 23	p>0.05	p>0.05	p>0.05	p>0.05	*
G"	5 vs 14	p>0.05	p>0.05	p>0.05	p>0.05	*
	5 vs 23	*	p>0.05	p>0.05	p>0.05	p>0.05
	14 vs 23	p>0.05	p>0.05	*	p>0.05	*
σ_0	5 vs 14	p>0.05	p>0.05	p>0.05	p>0.05	p>0.05
	5 vs 23	p>0.05	p>0.05	p>0.05	p>0.05	*
	14 vs 23	p>0.05	p>0.05	p>0.05	p>0.05	p>0.05
Droplet size	5 vs 14	*	*	*	p>0.05	*
	5 vs 23	*	*	*	*	*
	14 vs 23	p>0.05	p>0.05	p>0.05	*	p>0.05
IVRT Flux	5 vs 14	*	*	*	*	*
	5 vs 23	*	*	*	*	*
	14 vs 23	*	p>0.05	*	*	*
A_{6h}	5 vs 14	*	*	*	*	*
	5 vs 23	*	*	*	*	*
	14 vs 23	*	*	*	*	*
